# Predictive factors for risk of chronic evolution in Vogt–Koyanagi–Harada disease at 1 month after pulse steroid therapy

**DOI:** 10.1371/journal.pone.0362558

**Published:** 2026-11-11

**Authors:** Tetsuya Muto, Shoichiro Kusuda, Masaaki Sakamoto, Shigeki Machida, Shinichiro Imaizumi, Tetsuju Sekiryu

**Affiliations:** 1 Department of Ophthalmology, Dokkyo Medical University Saitama Medical Center, Koshigaya, Japan; 2 Imaizumi Eye Hospital, Koriyama, Japan; 3 Department of Ophthalmology, Fukushima Medical University, Fukushima, Japan; Max Planck Institute for Solid State Research, GERMANY

## Abstract

In this study, we aimed to identify factors for risk of chronic evolution in Vogt–Koyanagi–Harada (VKH) disease at 1 month following pulse steroid therapy. This was a retrospective clinical cohort, single center study. A total of 121 eyes from 61 treatment-naïve patients with acute VKH disease, who underwent pulse steroid therapy and had follow-up for over 12 months were evaluated. Patients were categorized into SGF and non-SGF groups according to fundus outcomes at 12 months after treatment. Anterior chamber morphology was assessed using Pentacam^®^, while axial length and lens thickness were measured with the IOLMaster 700. Multivariable analysis demonstrated that lower best-corrected visual acuity (BCVA) (*p* < 0.001) and increased corneal thickness (*p* = 0.016) at 1 month were significantly associated with the development of SGF. Receiver operating characteristic curve analysis determined optimal cut-off values for predicting SGF as a logarithmic BCVA greater than −0.040 and a corneal thickness exceeding 574.500 µm. The corresponding Youden index values were 0.291 for BCVA and 0.244 for corneal thickness. Elevated logarithmic BCVA values and increased corneal thickness at 1 month after treatment were identified as risk factors for the subsequent SGF development. Applying higher cut-off values for these factors may facilitate more targeted therapeutic strategies in patients with VKH disease.

## Introduction

Vogt–Koyanagi–Harada (VKH) disease is a bilateral granulomatous panuveitis with potential multisystem involvement. It is a T-cell-mediated autoimmune disorder that occurs in genetically susceptible individuals, in which cytotoxic T-cells target melanocytes [1]. As melanocytes are widely distributed throughout the body, systemic manifestations such as fever meningitis-like symptoms, headache, paresthesia of hair, and general malaise often precede the onset of ocular symptoms. The disease can develop across a wide age range, from infancy to older adulthood, but is reported more frequently in females [2–8]. Adequate inhibition of inflammation during the early stage of the disease is important for preventing recurrence and the persistence of inflammation during later progressive stages.

In Japan, corticosteroid pulse therapy consisting of 500–1000 mg of intravenous methylprednisolone administered over 3 days, followed by several months of oral corticosteroids, has been widely implemented for many years [2–8]. However, the recurrence rate of VKH disease remains approximately 25%, even with pulse steroid therapy [2–5]. Urzura et al. reported that chronic recurrent VKH disease is more refractory to treatment and is associated with a higher rate of complications [9]. Thus, strategies aimed at reducing disease recurrence are essential.

Several studies have indicated a strong association between disease recurrence and the development of sunset glow fundus (SGF) [2,3,6]. Thus, preventing SGF is an essential objective. El Asrar et al. showed that combining mycophenolate mofetil therapy with systemic corticosteroids was effective for reducing the risk of SGF [10].

We also recognized the importance of identifying predictive factors for the development of SGF [2]. Clarifying these predictive factors could allow for more effective treatment management. Hirooka et al. highlighted that central choroidal thickness, measured 1 week after systemic corticosteroid therapy, was a key indicator in treatment-naïve patients with VKH disease [6]. They reported a cut-off value of 410 μm for predicting SGF development. Although attempts have been made to identify predictive factors at the initial patient visit, the sensibility and specificity of these early indicators do not always have enough value [2].

The aim of this study was to identify factors quantifiable at 1 month after pulse steroid therapy, as well as at the initial patient visit, that could predict the risk of chronic evolution 12 months post-treatment in patients with acute VKH disease.

## Materials and methods

This retrospective clinical cohort study included 61 treatment-naïve patients with VKH disease totaling 121 eyes. The mean age of participants was 45.0 ± 14.3 years (range 13**–**77 years) at Dokkyo Medical University Saitama Medical Center between April 2015 and October 2024. All participants were followed up for over 12 months after initiating pulse steroid therapy.

The type of VKH disease was classified according to recent diagnostic criteria [11]. The older revised diagnostic criteria [12] may have certain disadvantages because recent criteria have higher diagnostic sensitivity [11].

Patients diagnosed at the initial visit with chronic VKH disease, amblyopia, senile cataract, senile dementia, ischemic optic neuropathy, high myopia (less than −8 diopters), or hypermetropia (greater than +5 diopters) were excluded from this study.

Pulse steroid therapy was initiated with intravenous methylprednisolone (Solu-Medrol; Pfizer, NY, USA) at a dose of 1,000 mg/day for 3 days, followed by oral prednisolone (Predonine; Shionogi, Osaka, Japan). Oral prednisolone was started at 40 mg/day and tapered by 5 mg/day every 3 − 4 weeks, as previously described [2,3]. Although one patient was 13 years old, her body weight (48 kg) allowed for the administration of the standard steroid regimen. Patients with recurrence limited to the anterior segment were treated with 0.1% fluorometholone ophthalmic suspension (Santen, Osaka, Japan) or Sanbetason ophthalmic and otorhinologic solution (Santen, Osaka, Japan). Patients with recurrence in the posterior segment were treated with oral corticosteroids or sub-Tenon triamcinolone acetonide injections. If anterior inflammation progressed to the chronic type and became resistant to steroid therapy, adalimumab was administered.

During therapy, secondary cataract development was limited to the posterior subcapsular forms and affected best-corrected visual acuity (BCVA).

Written informed consent for treatment was obtained from all participants. The study protocol adhered to the Declaration of Helsinki and was approved by the Institutional Review Board of Dokkyo Medical University Saitama Medical Center (#25114). Informed consent for publication was waived due to the retrospective design of the study. The data were accessed for research purposes on March 28, 2026

### Biomarker measurement

At the initial visit, all patients underwent comprehensive ophthalmologic evaluations, including standardized refraction, BCVA measurement using a Landolt ring, slit-lamp biomicroscopy, anterior segment analysis, axial length (AL) measurement, optical coherence tomography (OCT), and color fundus photography. Decimal BCVA values were converted to the logarithm of the minimal angle of resolution.

Fluorescein angiography (FAG) and indocyanine green angiography (ICGA) were performed on 54 patients. Four patients did not undergo urgent FAG or ICGA because recent angiographic records from previous hospitalizations were available, while three patients did not undergo the procedures due to emergency admissions. Refractive status was assessed using an autorefractometer (KR-7100; Topcon Corp., Tokyo, Japan), and the spherical equivalent (SE) was calculated as the spherical power plus half of the cylindrical power.

Anterior chamber parameters, including volume (ACV), central depth (ACD), peripheral depth, angle (ACA), corneal thickness, and pupil diameter were measured using Pentacam^Ⓡ^ software. All anterior segment measurements were obtained with a contracted pupil. Peripheral ACD was calculated as the mean of four points located 3 mm from the pupil center. AL and lens thickness were measured using the IOLMaster 700 (Carl Zeiss Meditec AG, Jena, Germany). Retinal morphology was assessed using spectral-domain OCT (RS-3000 Advance, Nidek Corp., Japan), with scans obtained through both horizontal and vertical foveal passes. Average retinal foveal thickness (RFT) was calculated as the mean of these horizontal and vertical thickness measurements. Swept-source OCT (SS-OCT; PLEX Elite 9000, Carl Zeiss Meditec AG, Jena, Germany) was used to measure choroidal foveal thickness (CFT), defined as the distance from the outer border of the retinal pigment epithelium to the inner scleral border at the fovea. Values exceeding 800 μm were limited by image resolution. All OCT images were independently analyzed by a blinded author (M.S.).

A single investigator (TM) assessed the ocular findings at approximately monthly intervals, and routinely determined the onset of SGF based on the appearance of the orange-red fundus discoloration that had not been present at the visit 1 month earlier. The presence of SGF in each eye was subsequently reconfirmed by reviewing the color fundus photographs.

Patients were stratified into two groups based on the presence or absence of SGF at 12 months, designated as the SGF and a non-SGF groups. Correlations between measurements obtained at the initial visit and at 1-month, as well as their associations with fundus outcomes at 12-months, were analyzed.

### Statistical analysis

Descriptive data are presented as means ± standard deviations. To evaluate the statistical significance of changes over time after the initiation of pulse steroid therapy, a one-way repeated-measures ANOVA was conducted, followed by Tukey’s post hoc tests. Generalized estimating equations (GEEs) were used, with Bonferroni correction applied for multiple comparisons. The unpaired t-test was used to compare the SGF and non-SGF groups at each time point. Stepwise multivariable analysis, with a *p*-value threshold of 0.2, was applied to identify factors associated with 12-month fundus outcomes. These outcomes included BCVA, ACV, ACD (also peripheral), corneal thickness, SE and changes in these parameters from the initial visit to 1 month, difference of ACV from initial visit to 1 month post-treatment, as well as ACA, pupil diameter, AL, RFT, and CFT. StatMate version V for Macintosh (ATMS, Tokyo, Japan) was used to perform one-way repeated-measures ANOVA, Tukey’s tests, unpaired *t*-tests, and chi-square tests. JMP 16 Pro (SAS Institute, Inc., Cary, NC, USA) was employed for multivariable analysis, and IBM SPSS Statistics 29.0.0 (IBM Corp., Tokyo, Japan) was used for receiver operating characteristic (ROC) analysis, GEEs and Bonferroni adjusted comparisons. Optimal cut-off values in ROC analyses were determined using the Youden index. Statistical significance was set at *p* < 0.05.

## Results

Table 1 presents the demographic characteristics of patients in the SGF and non-SGF groups at the initial visit. No significant differences were observed between groups for any demographic factors.

**Table 1:** Demographic characteristics of patients with VKH at the initial visit, categorized by subsequent SGF development.

	SGF	Non-SGF	*p* value
***At the initial visit in 61 patients***			
Age (years)	46.1 ± 12.8	43.8 ± 16.5	0.56
Gender (male/female)	15/17	14/15	0.91
Body weight (kg)	63.6 ± 14.7	61.0 ± 13.0	0.48
From symptom onset to initial visit (days)	19.3 ± 42.8	10.1 ± 7.9	0.24
Meningismus (yes/no)	24/8	23/6	0.69
Recent revised diagnostic criteria (variant 1/variant 2)	28/4	23/6	0.61
Smoking condition			
(smoker/past smoker/non-smoker)	11/2/19	8/7/14	0.14
Synechia between lens and iris (yes/no)	10/53	4/54	0.21
HbA1c (%)	5.91 ± 1.39	5.59 ± 0.43	0.22

Abbreviations: HbA1c, glycated hemoglobin; SGF, sunset glow fundus.

Table 2 summarizes the clinical characteristics of 121 eyes from 61 patients who were followed for more than 12 months after pulse steroid therapy. The non-SGF group had fewer cases of secondary cataracts and a lower disease recurrence rate within 12 months than the SGF group. No significant differences in BCVA were observed between the groups.

**Table 2:** Clinical characteristics of patients observed for >12 months after pulse steroid therapy in the SGF and non-SGF groups.

	SGF	Non-SGF	*p* value
***At 12 months in 61 patients***			
BCVA at 12 months	–0.069 ± 0.042	–0.043 ± 0.094	0.053
Secondary cataract (yes/no)	6/57	0/58	0.046
Recurrence experience (yes/no)	13/19	2/27	<0.01

Abbreviations: BCVA, best-corrected visual acuity; SGF, sunset glow fundus.

In the multivariate analysis, lower BCVA (*p* < 0.001) and greater cornea thickness (*p* = 0.016), were significantly associated with SGF at 12 months. Table 3 shows the cut-off values for BCVA and corneal thickness, determined by ROC curve analysis for SGF at 12 months. Although the sensitivity values for both factors were below 0.5, the specificity values exceeded 0.862. The corresponding Youden index values for each factor were less than 0.3.

**Table 3:** Cut-off values determined by ROC curves for SGF at 12 months.

	Cut-off value	Sensitivity	Specificity	Youden index
BCVA	–0.040	0.429	0.862	0.291
Corneal thickness (µm)	574.5	0.365	0.879	0.244

Abbreviations: BCVA, best-corrected visual acuity.

Fig 1 illustrates the chronological changes in average biomarker levels in both groups. The biomarkers included BCVA, anterior-chamber parameters, SE, AL, lens thickness, RFT, and CFT after the initiation of pulse steroid therapy. BCVA values increased significantly over time course in both groups (*p* < 0.001) according to one-way repeated-measures ANOVA (Fig 1a). ACV in the SGF group also increased significantly over time (*p* < 0.01), whereas ACV in the non-SGF group remained unchanged (*p* = 0.16) (Fig 1b). Central ACD values increased significantly over time in both groups (SGF group: *p* < 0.001, non-SGF group: *p* < 0.01) (Fig 1c). Peripheral ACD showed a similar pattern (SGF group: *p* < 0.001, non-SGF group: *p* < 0.05) (Fig 1d). ACA increased significantly increased in the SGF group (*p* < 0.05), but remained largely unchanged in the non-SGF group (*p* = 0.062) (Fig 1e). Pupil diameter decreased significantly over time in both groups (*p* < 0.05) (Fig 1f). Corneal thickness declined significantly over time in the SGF group (*p* < 0.05) but remained stable in the non-SGF group (*p* = 0.71) (Fig 1g). A similar trend was observed for SE in the non-SGF group, while the SGF group showed no significant changes (*p* = 0.40) (Fig 1h). AL increased significantly in the SGF group (*p* < 0.05) but showed no significant change in the non-SGF group (*p* = 0.089) (Fig 1i). Lens thickness remained stable over time (SGF group: *p* = 0.54, non-SGF group: *p* = 0.74) (Fig 1j). Both RFT and CFT decreased significantly over the observation period in both groups according to one-way repeated-measures ANOVA (*p* < 0.001) (Fig 1k and 1l).

**Fig 1:**
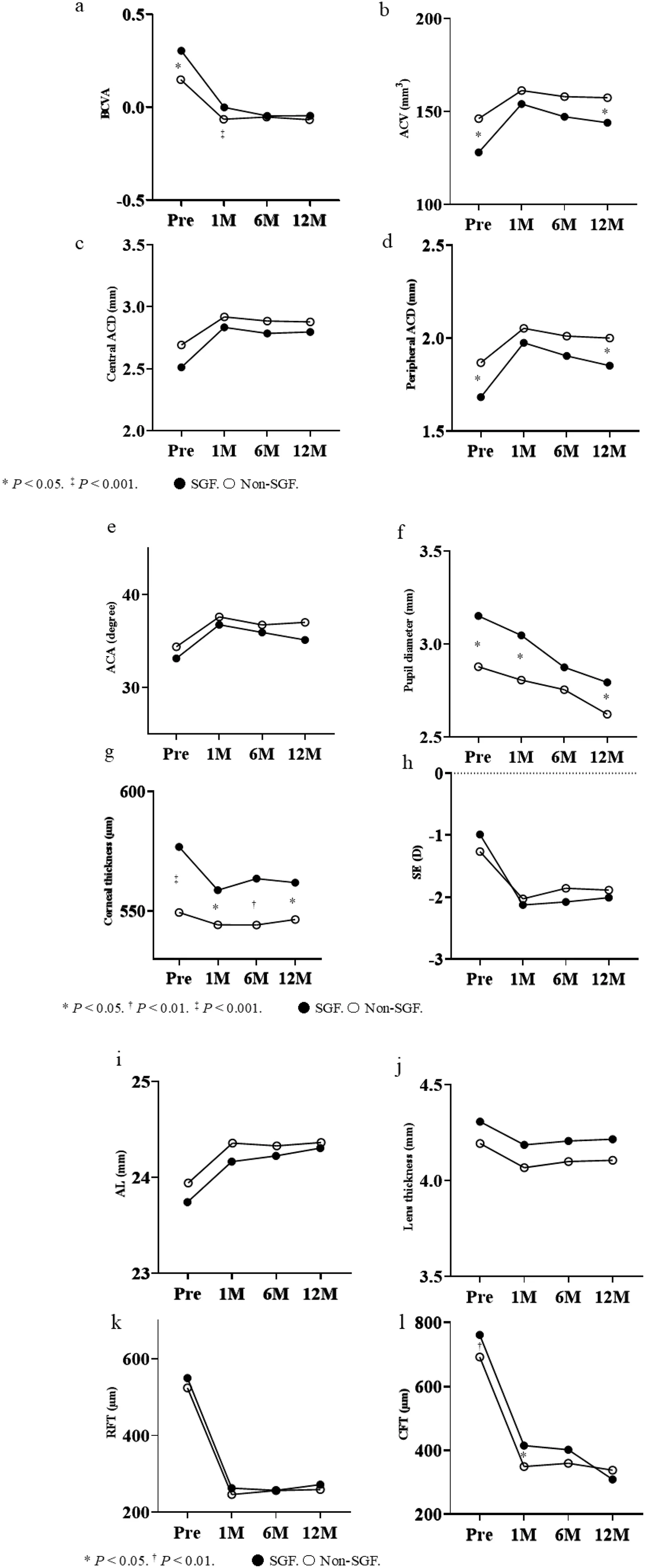
(a) Longitudinal changes in BCVA, as the logarithm of the minimal angle of resolution for both the SGF and non-SGF groups at pretreatment and months 1, 6, and 12, with significant differences at pretreatment (*p* < 0.05) and month 1 (*p* < 0.001). **(b)** ACV at pretreatment and 1, 6, and 12 months with significant differences at pretreatment (*p* < 0.05) and month at 12 months post-treatment (*p* < 0.05). **(c)** Central ACD at pretreatment and at 1, 6, and 12 months post-treatment, without significant differences. **(d)** Peripheral ACD at pretreatment and at 1, 6, and 12 months post-treatment, with significant differences at pretreatment (*p* < 0.05) and 12 months (*p* < 0.05). **(e)** ACA at pretreatment and at 1, 6, and 12 months post-treatment, without significant differences. **(f)** Pupil diameter at pretreatment and at 1, 6, and 12 months post-treatment, with significant differences at pretreatment (*p* < 0.05), 1 month (*p* < 0.05), and 12 months (*p* < 0.05). **(g)** Corneal thickness at pretreatment and at 1, 6, and 12 months post-treatment, showing significant differences at pretreatment (*p* < 0.001) and at 1 month (*p* < 0.05), 6 months (*p* < 0.01), and 12 months (*p* < 0.05). (h) SE was at pretreatment and at 1, 6, and 12 without significant difference. **(i)** AL at pretreatment and at 1, 6, and 12 months post-treatment, without significant differences. **(j)** Lens thickness at pretreatment and at 1, 6, and 12 months post-treatment, without significant differences. **(k)** RFT at pretreatment and at 1, 6, and 12 months post-treatment, without significant differences according to the unpaired t-test. **(l)** CFT at pretreatment and at 1, 6, and 12 months post-treatment, with significant differences at pretreatment (*p* < 0.01), and 1 month (*p* < 0.05) according to the unpaired t-test. Abbreviations: ACA, anterior chamber angle; ACD, anterior chamber depth; ACV, anterior chamber volume; AL, axial length; BCVA, best-corrected visual acuity; CFT, choroidal foveal thickness; RFT, retinal foveal thickness; SE, spherical equivalent.

Fig 2 illustrates the chronological changes in mean biomarker values in both groups, as analyzed using GEEs. BCVA improved significantly over time in both groups (Fig 2a). ACV (Fig 2b) and central ACD values (Fig 2c) also increased significantly over time in both groups. Peripheral ACD showed a similar pattern (Fig 2d). ACA increased significantly in the non-SGF group remained largely unchanged in the SGF group (Fig 2e). Pupil diameter (Fig 2f) and corneal thickness (Fig 2g) remained unchanged over time in both groups. SE decreased significantly in the SGF group, but showed minimal change in the non-SGF group (Fig 2h). AL increased significantly in both groups (Fig 2i). Lens thickness decreased significantly over time in the non-SGF group, but remained stable in the SGF group (Fig 2j). Both RFT and CFT decreased significantly over the observation period in both groups according to the GEE analysis (*p* < 0.001) (Fig 2k and 2l).

**Fig 2:**
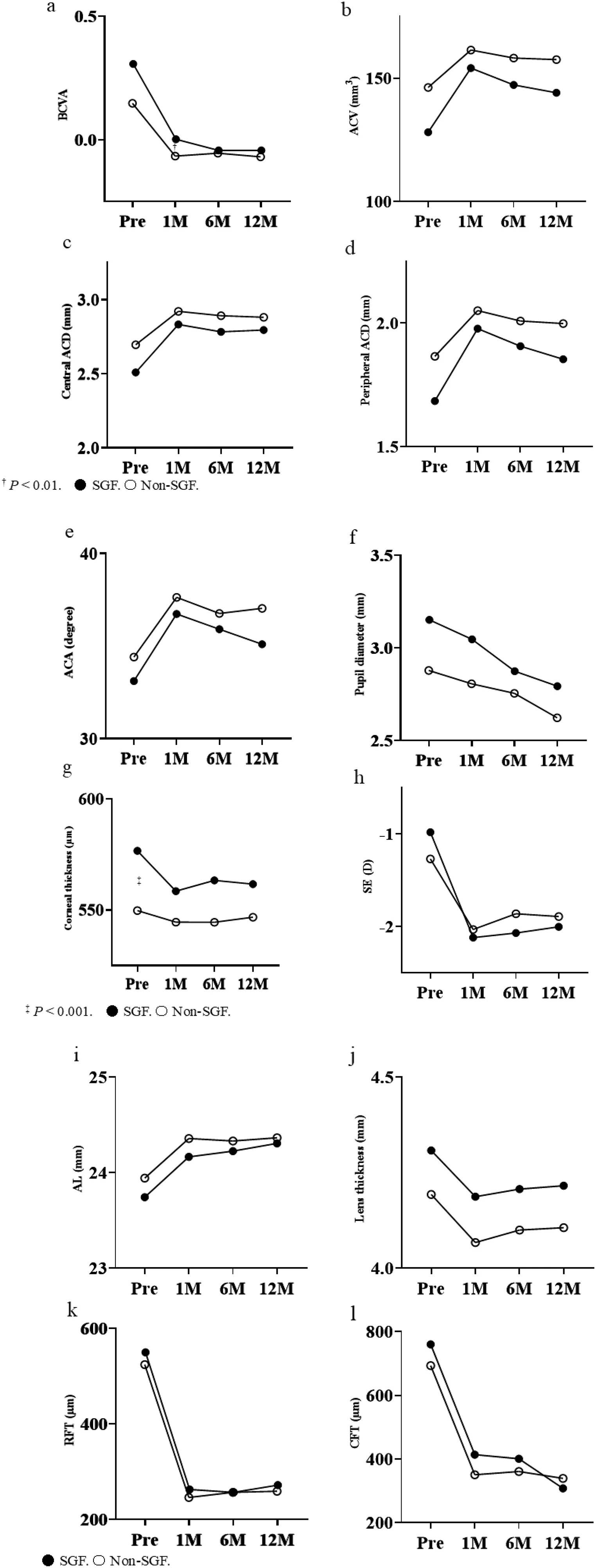
(a) Longitudinal changes in BCVA, as the logarithm of the minimum angle of resolution for the SGF and non-SGF groups at pretreatment and at 1, 6, and 12 months post-treatment. A significant difference was observed at 1 month (*p* < 0.01). **(b)** ACV at pretreatment and at 1, 6, and 12 months post-treatment. without significant differences. **(c)** Central ACD at pretreatment and at 1, 6, and 12 months post-treatment. No significant differences were observed. **(d)** Peripheral ACD at pretreatment and at 1, 6, and 12 months post-treatment. No significant differences were observed. **(e)** ACA at pretreatment and at 1, 6, and 12 months post-treatment. No significant differences were observed. **(f)** Pupil diameter at pretreatment and at 1, 6, and 12 months post-treatment. No significant differences were observed. **(g)** Corneal thickness at pretreatment and at 1, 6, and 12 months post-treatment. Significant differences were observed at pretreatment (*p* < 0.001). (h) SE at pretreatment and at 1, 6, and 12 months post-treatment. No significant differences were observed. **(i)** AL at pretreatment and at 1, 6, and 12 months post-treatment. No significant differences were observed. **(j)** Lens thickness at pretreatment and at 1, 6, and 12 months post-treatment. No significant differences were observed. **(k)** RFT at pretreatment and at 1, 6, and 12 months post-treatment. No significant differences were observed. **(l)** CFT at pretreatment and at 1, 6, and 12 months post-treatment. No significant differences were observed. Abbreviations: ACA, anterior chamber angle; ACD, anterior chamber depth; ACV, anterior chamber volume; AL, axial length; BCVA, best-corrected visual acuity; CFT, choroidal foveal thickness; RFT, retinal foveal thickness; SE, spherical equivalent.

Fig 3 presents a comparative analysis of BCVA and corneal thickness between the SGF and non-SGF groups. The cut-off values for these parameters were determined using ROC analysis (Fig 3a and 3b).

**Fig 3:**
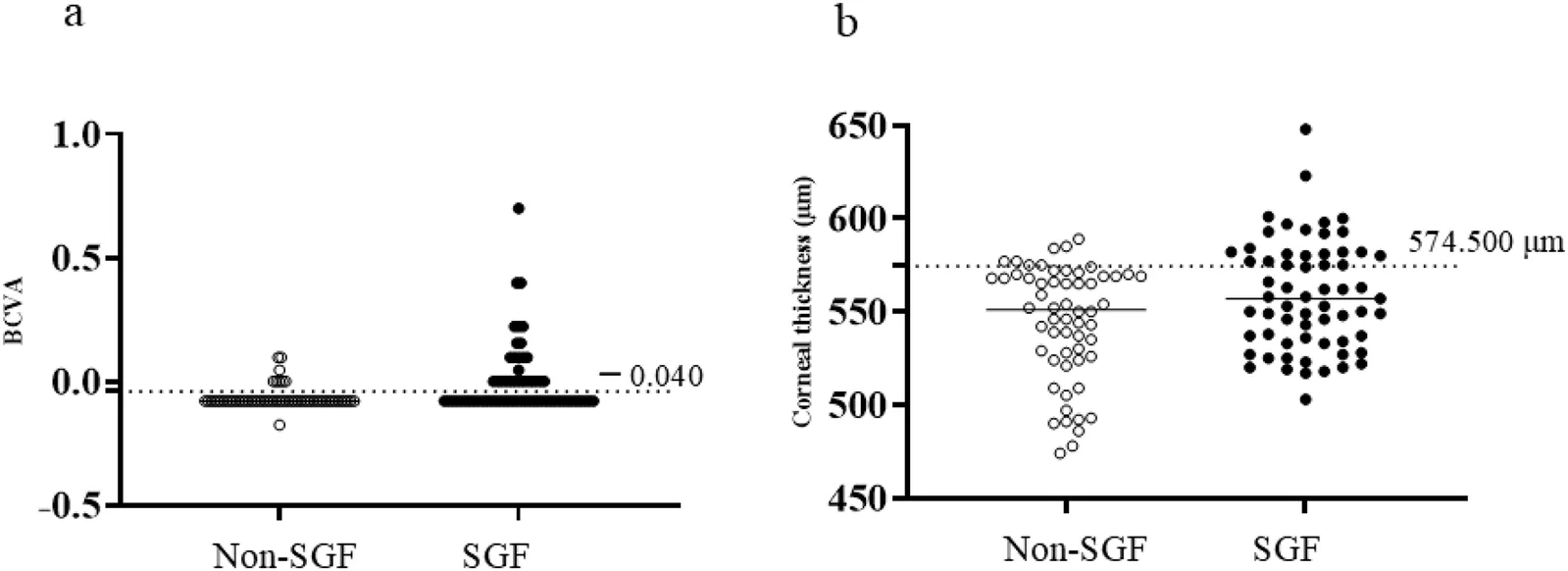
(a) Comparison of BCVA between the SGF and non-SGF groups at 1 month after pulse steroid therapy. The determined cut-off value for BCVA was –0.040. **(b)** Comparison of corneal thickness between the groups at 1 month post-treatment. The cut-off value for corneal thickness was 574.500 μm between the two groups. Abbreviations: BCVA, best-corrected visual acuity; SGF, sunset glow fundus.

## Discussion

In our previous study, we identified predictive factors for SGF at the initial visit [2]. However, these factors did not consistently show high sensitivity and specificity [2]. In this study, we aimed to identify more reliable predictive factors with higher diagnostic values. Using data collected at the initial visit and 1 month post-treatment, we identified only two factors with a Youden index below 0.3. Although these two factors may have some clinical utility, they have limited predictive power. Future research should focus on identifying predictive factors with a Youden index exceeding 0.5 to provide stronger clinical guidance. The differences observed between these time points appeared to be significant in the SGF group, as illustrated in Fig 1a–1d, 1g, and 1h. Based on these observations, we conducted multivariate analyses of the changes in BCVA, ACV, central and peripheral ACD, corneal thickness, and SE. However, none of these changes were significantly associated with SGF.

Corneal thickness at the initial visit and 1 month post-treatment was associated with SGF in the previous [2] and present studies, respectively. To the best of our knowledge, only two reports have examined the relationship between these two parameters [2,13], based on searched of the PubMed database (key words: “Vogt–Koyanagi–Harada” “corneal thickness”; search years, 1950–2025) and the CiNii Research database (keywords: “Vogt–Koyanagi–Harada”, “corneal thickness”; search years, 1990–2025). Hamano et al. reported that central corneal thickness decreased significantly after treatment and correlated with flare values, as measured by anterior SS-OCT (CASIA-2) [13]. These findings suggest that the association between VKH disease and corneal morphology is clinically important, and further research may yield additional insights in this field.

No previous studies have reported an association between SGF and visual acuity measured at 1 month after therapy, based on searches of the PubMed database (keywords: “Vogt–Koyanagi–Harada,” “sunset glow fundus,” “visual acuity”; search years: 1950–2025) and the CiNii Research database (keywords: “Vogt–Koyanagi–Harada,” “sunset glow fundus,” “visual acuity”; search years: 1990–2025). However, several reports have documented such associations at the initial visit [14–16]. Future studies are warranted to examine the relationships between SGF development and visual acuity measured on day 1 and weeks 1 and 2 after therapy.

The Youden index, defined as max_c_ {*sensitivity(c)+specificity(c)*-1}, is one of the most widely used metrics because it integrates naturally within the ROC framework [17]. Its values range from 0 to 1, with a value of 1 indicating perfect discriminatory performance, while a value of 0 indicates that the biomarker performs no better than chance [18]. The clinical relevance of the Youden index is well illustrated in the context of adenoviral conjunctivitis. Morozumi et al. reported sensitivity and specificity values exceeding 90% for a recently developed immunochromatographic assay for adenovirus detection [19], indicating excellent diagnostic performance. In the future studies, we aim to identify SGF-related predictors in patients with VKH disease that achieve comparable levels of accuracy.

Based on our findings, we identified, to a certain extent, cases with a higher likelihood of developing SGF. Establishing an appropriate management method for such cases is therefore clinically important. However, it is uncertain whether the simple increase in corticosteroid dosage, the addition of sub-tenon triamcinolone acetonide injection [20], combination therapy with an immunosuppressive agent [7,10,20,21] or biologicals [14,22] provides the greatest benefit. Future studies are needed to clarify the most effective treatment method and to determine whether optimized management can reduce SGF incidence We hope coping methods will be made clear in the future and reduce SGF incidence.

It is now well recognized that adequate of early immunosuppression plays a critical role in preventing the chronic evolution of VKH disease. Tsubota et al. reported no significant difference in clinical efficacy between conventional pulse steroid therapy (1,000 mg/day of intravenous methylprednisolone for 3 days) and half-pulse steroid therapy (500 mg/day of intravenous methylprednisolone for 3 days) for VKH disease [8], concluding that half-pulse steroid therapy may reduce the risk of corticosteroid-related adverse effects [8]. They demonstrated that differences in initial steroid dosing strategies can significantly influence clinical outcomes, highlighting the importance of treatment-related variables in determining long-term disease progression [8]. Because pulse steroid therapy is associated with several adverse effects [23], their study is noteworthy in suggesting a strategy to reduce this risk [8].

Yang et al. reported that meningismus, tinnitus, dysacusis, and high sensitivity to touch of the hair were observed in 42.3%, 30.2%, 7.5%, and 6.6% of the pediatric VKH patients (age of ≤ 16 years), respectively, whereas a significantly higher proportion of these manifestations was observed in adult (16 <age < 65 years) and elderly (age of ≥ 65 years) VKH patients (all *p* < 0.001) [24]. Furthermore, they concluded that the pediatric or elderly VKH patients showed a favourable visual prognosis, whereas the adult VKH patients were associated with a higher risk of poor visual outcomes [24]. Thus, extraocular manifestations might have been strongly associated with VKH disease progression [24,25]. However, history-taking was insufficient, and only meningismus was confirmed in all patients. This study focused exclusively on ocular parameters. If history-taking were sufficient, this study would be more informative.

The present study has several limitations. First, patients were classified into the SGF and non-SGF groups on the basis of fundus findings 12 months after treatment initiation. In clinical practice, a definitive diagnosis of SGF often requires observation beyond 12 months, particularly in patients who receive aggressive early immunosuppressive therapy. Therefore, classification of the SGF and non-SGF groups based on fundus findings obtained at a later time point may be more appropriate. Second, we assessed predictive factors at 1 month after pulse steroid therapy. At this stage, patients are still in the early recovery phase of acute VKH disease, and inflammatory activity may fluctuate depending on treatment response, the tapering schedule, and individual immune dynamics. Therefore, it may be unrealistic to assume that a single measurement obtained at this time point can serve as a reliable predictor of a chronic structural outcome such as SGF. Third, the Youden index values for both BCVA and corneal thickness were <0.3, and the sensitivity values for both predictors were <0.5, as shown in Table 3. These findings suggest that the proposed predictors have limited clinical utility. Fourth, corneal thickness may represent a nonspecific marker of systemic or ocular inflammation rather than a disease-specific predictor. It was not possible to distinguish between these possibilities in the present study. Fifth, because we did not perform an a priori sample size calculation, the possibility of insufficient statistical power and the absence of significant differences cannot be excluded. To further assess the adequacy of the sample size, we performed a post hoc power analysis using the R software [26]. Based on the Youden index threshold of 0.5 and specificity of 0.862 observed in the present study, the sensitivity was calculated to be 0.638 using the following formula: Youden index = sensitivity + specificity − 1 [17]. Based on these estimates, the required sample size was calculated to be 538 eyes. Although this post hoc analysis does not replace an a priori sample size calculation, it provides additional information regarding the sample size adequacy in this study. Thus, further well-designed prospective clinical studies with an appropriate a priori sample size calculation are needed to validate our findings.

In conclusion, this study identified two risk factors at 1 month post-treatment, along with their corresponding cut-off values, that are associated with the risk of chronic evolution. These findings may support more informed clinical decision-making in patients with VKH disease, particularly those presenting with BCVA and corneal thickness values exceeding the identified thresholds. Our future objectives include identifying more reliable predictive factors and establishing management strategies for cases exceeding the identified cut-off values.
