# Supplementary material for: Predictive factors for risk of chronic evolution in Vogt–Koyanagi–Harada disease at 1 month after pulse steroid therapy
Source: PLoS One. 2026 Nov 11;21(11):e0362558. doi: 10.1371/journal.pone.0362558 (PMC13754166; doi:10.1371/journal.pone.0362558)
Supplement: Supplementary file 2 [file pone.0362558.s003.docx]

Review Comments to the Author

Please use the space provided to explain your answers to the questions above. You may also include additional comments for the author, including concerns about dual publication, research ethics, or publication ethics. (Please upload your review as an attachment if it exceeds 20,000 characters)

Reviewer #2: The authors have addressed all the major issues of the original submission, and the current version is acceptable to be published.

Reviewer #3: Comments to the Authors:
The manuscript investigates early predictive factors for sunset glow fundus (SGF) development in patients with acute VKH disease. While the study addresses a clinically relevant question, several methodological and conceptual concerns must be addressed before the manuscript can be considered for publication.
Major Concerns:
1. Role of Extraocular Manifestations: The current study focuses exclusively on ocular parameters (anterior chamber morphology, BCVA, etc.). However, systemic manifestations, such as meningismus, tinnitus, dysacusis, and high sensitivity to touch of the hair, are frequently observed in VKH disease and have been strongly associated with disease progression. The authors should analyze the role of extraocular manifestations in their cohort as potential predictors for SGF. (PMID: 37058172; 17123618).

**Yes, we have added the following paragraph in discussion section. According to this revision, we have cited references no. 24 and 25. Furthermore, we revised Table 1 as below.**

Yang et al. reported that meningismus, tinnitus, dysacusis, and high sensitivity to touch of the hair were observed in 42.3%, 30.2%, 7.5%, and 6.6% of the pediatric VKH patients (age of ≤ 16 years), respectively, whereas a significantly higher proportion of these manifestations was observed in adult (16 < age < 65 years) and elderly (age of ≥ 65 years) VKH patients (all *p* < 0.001) [24]. Furthermore, they concluded that the pediatric or elderly VKH patients showed a favourable visual prognosis, whereas the adult VKH patients were associated with a higher risk of poor visual outcomes [24]. Thus, extraocular manifestations might have been strongly associated with VKH disease progression [24, 25]. However, history-taking was insufficient, and only meningismus was confirmed in all patients. This study focused exclusively on ocular parameters. If history-taking were sufficient, this study would be more informative.

24. Yang P, Liao W, Pu Y, Zhong Z, Wang H, Yu Q, et al. Vogt-Koyanagi-Harada disease in pediatric, adult and elderly: clinical characteristics and visual outcomes. Graefes Arch Clin Exp Ophthalmol. 2023;261:2641–2650. doi: 10.1007/s00417-023-06058-5.
25. Yang P, Ren Y, Li B, Fang W, Meng Q, Kijlstra A. Clinical characteristics of Vogt-Koyanagi-Harada syndrome in Chinese patients. Ophthalmology. 2007;114:606–614. doi: 10.1016/j.ophtha.2006.07.040.

**Table 1.** Demographic Characteristics of Patients with VKH at the Initial Visit, Categorized by Subsequent SGF Development

|  | SGF | Non-SGF | *p* value |
| --- | --- | --- | --- |
| ***At the initial visit in 61 patients*** |  |  |  |
| Age (years) | 46.1 ± 12.8 | 43.8 ± 16.5 | 0.56 |
| Gender (male/female) | 15/17 | 14/15 | 0.91 |
| Body weight (kg) | 63.6 ± 14.7 | 61.0 ± 13.0 | 0.48 |
| From symptom onset to initial visit (days) | 19.3 ± 42.8 | 10.1 ± 7.9 | 0.24 |
| Meningismus (yes/no) | 24/8 | 23/6 | 0.69 |
| Recent revised diagnostic criteria  (variant 1/variant 2) | 28/4 | 23/6 | 0.61 |
| Smoking condition |  |  |  |
| (smoker/past smoker/non-smoker) | 11/2/19 | 8/7/14 | 0.14 |
| Synechia between lens and iris (yes/no) | 10/53 | 4/54 | 0.21 |
| HbA1c (%) | 5.91 ± 1.39 | 5.59 ± 0.43 | 0.22 |

Abbreviations: HbA1c, glycated hemoglobin; SGF, sunset glow fundus.

2. The manuscript does not clearly state which diagnostic criteria were used to classify the patients at the initial visit. While older criteria are traditionally common, recent literature has introduced updated diagnostic criteria for VKH disease that demonstrate significant advantages, notably a higher diagnostic sensitivity (PMID: 29978190). Please clarify the exact diagnostic criteria applied to this cohort in the Methods section.

**Yes, we have added the following paragraph in materials and methods section. According to this revision, we have cited references no. 11 and 12.**

The type of VKH disease was classified according to recent diagnostic criteria [11]. The older revised diagnostic criteria [12] may have certain disadvantages because recent criteria have higher diagnostic sensitivity [11].

11. Yang P, Zhong Y, Du L, Chi W, Chen L, Zhang R, et al. Development and evaluation of diagnostic criteria for Vogt-Koyanagi-Harada disease. JAMA Ophthalmol. 2018;136:1025–1031. doi: 10.1001/jamaophthalmol.2018.2664.
12. Read RW, Holland GN, Rao NA, Tabbara KF, Ohno S, Arellanes-Garcia L, et al. Revised diagnostic criteria for Vogt-Koyanagi-Harada disease: report of an international committee on nomenclature. Am J Ophthalmol. 2001;131:647–652. doi: 10.1016/s0002-9394(01)00925-4.

3. The study cohort consists of 61 patients (121 eyes). It is unclear if this sample size provides sufficient statistical power to support the conclusions drawn from the multivariable analysis, particularly given the low sensitivity (<0.5) and Youden index (<0.3) values reported. Has a formal a priori sample size calculation or post hoc power analysis been performed? Please clarify this in the methodology.

**Yes, we have added the following sentences in discussion section . According to this revision, we have cited reference no. 26.**

The present study has several limitations. First, patients were classified into the SGF and non-SGF groups on the basis of fundus findings 12 months after treatment initiation. In clinical practice, a definitive diagnosis of SGF often requires observation beyond 12 months, particularly in patients who receive aggressive early immunosuppressive therapy. Therefore, classification of the SGF and non-SGF groups based on fundus findings obtained at a later time point may be more appropriate. Second, we assessed predictive factors at 1 month after pulse steroid therapy. At this stage, patients are still in the early recovery phase of acute VKH disease, and inflammatory activity may fluctuate depending on treatment response, the tapering schedule, and individual immune dynamics. Therefore, it may be unrealistic to assume that a single measurement obtained at this time point can serve as a reliable predictor of a chronic structural outcome such as SGF. Third, the Youden index values for both BCVA and corneal thickness were < 0.3, and the sensitivity values for both predictors were < 0.5, as shown in Table 3. These findings suggest that the proposed predictors have limited clinical utility. Fourth, corneal thickness may represent a nonspecific marker of systemic or ocular inflammation rather than a disease-specific predictor. It was not possible to distinguish between these possibilities in the present study. Fifth, because we did not perform an a priori sample size calculation, the possibility of insufficient statistical power and the absence of significant differences cannot be excluded. To further assess the adequacy of the sample size, we performed a post hoc power analysis using the R software [26]. Based on the Youden index threshold of 0.5 and specificity of 0.862 observed in the present study, the sensitivity was calculated to be 0.638 using the following formula: Youden index = sensitivity + specificity − 1 [17]. Based on these estimates, the required sample size was calculated to be 538 eyes. Although this post hoc analysis does not replace an a priori sample size calculation, it provides additional information regarding the sample size adequacy in this study. Thus, further well-designed prospective clinical studies with an appropriate a priori sample size calculation are needed to validate our findings.

26. R Core Team. R: A Language and Environment for Statistical Computing. R Foundation for Statistical Computing, Vienna, Austria. 2025. Available from: <https://www.R-project.org/>
