# Supplementary material for: Predictive factors for risk of chronic evolution in Vogt–Koyanagi–Harada disease at 1 month after pulse steroid therapy
Source: PLoS One. 2026 Nov 11;21(11):e0362558. doi: 10.1371/journal.pone.0362558 (PMC13754166; doi:10.1371/journal.pone.0362558)
Supplement: Peer review history

# Peer review: Decision Letter 0

**DOI:** 10.1371/journal.pone.0362558.r001

**Authors:** Robin Haunschild

26 Apr 2026

Dear Dr. Muto,

Thank you for submitting your manuscript to PLOS ONE. After careful consideration, we feel that it has merit but does not fully meet PLOS ONE’s publication criteria as it currently stands. Therefore, we invite you to submit a revised version of the manuscript that addresses the points raised during the review process.

Please submit your revised manuscript by Jun 10 2026 11:59PM. If you will need more time than this to complete your revisions, please reply to this message or contact the journal office at plosone@plos.org. When you're ready to submit your revision, log on to https://www.editorialmanager.com/pone/ and select the 'Submissions Needing Revision' folder to locate your manuscript file.

- A letter that responds to each point raised by the academic editor and reviewer(s). You should upload this letter as a separate file labeled 'Response to Reviewers'.
- A marked-up copy of your manuscript that highlights changes made to the original version. You should upload this as a separate file labeled 'Revised Manuscript with Track Changes'.
- An unmarked version of your revised paper without tracked changes. You should upload this as a separate file labeled 'Manuscript'.

If you would like to make changes to your financial disclosure, please include your updated statement in your cover letter. Guidelines for resubmitting your figure files are available below the reviewer comments at the end of this letter.

If applicable, we recommend that you deposit your laboratory protocols in protocols.io to enhance the reproducibility of your results. Protocols.io assigns your protocol its own identifier (DOI) so that it can be cited independently in the future. For instructions see: https://journals.plos.org/plosone/s/submission-guidelines#loc-laboratory-protocols. Additionally, PLOS ONE offers an option for publishing peer-reviewed Lab Protocol articles, which describe protocols hosted on protocols.io. Read more information on sharing protocols at https://plos.org/protocols?utm_medium=editorial-email&utm_source=authorletters&utm_campaign=protocols.

As the corresponding author, your ORCID iD is verified in the submission system and will appear in the published article. PLOS supports the use of ORCID, and we encourage all coauthors to register for an ORCID iD and use it as well. Please encourage your coauthors to verify their ORCID iD within the submission system before final acceptance, as unverified ORCID iDs will not appear in the published article. *Only*  the individual author can complete the verification step; PLOS staff *cannot*  verify ORCID iDs on behalf of authors.

We look forward to receiving your revised manuscript.

Kind regards,

Robin Haunschild

Academic Editor

PLOS One

Journal Requirements:

When submitting your revision, we need you to address these additional requirements.

1. Please ensure that your manuscript meets PLOS ONE's style requirements, including those for file naming. The PLOS ONE style templates can be found at

https://journals.plos.org/plosone/s/file?id=wjVg/PLOSOne_formatting_sample_main_body.pdf and

https://journals.plos.org/plosone/s/file?id=ba62/PLOSOne_formatting_sample_title_authors_affiliations.pdf

2. Thank you for stating the following in the Acknowledgments Section of your manuscript:

“The authors received no external funding or financial support.”

We note that you have provided funding information that is not currently declared in your Funding Statement. However, funding information should not appear in the Acknowledgments section or other areas of your manuscript. We will only publish funding information present in the Funding Statement section of the online submission form.

Please remove any funding-related text from the manuscript and let us know how you would like to update your Funding Statement. Currently, your Funding Statement reads as follows:

“The author(s) received no specific funding for this work.”

Please include your amended statements within your cover letter; we will change the online submission form on your behalf.

3. Please include a separate caption for each figure in your manuscript.

4. We note that Figure 1 in your submission contain copyrighted images. All PLOS content is published under the Creative Commons Attribution License (CC BY 4.0), which means that the manuscript, images, and Supporting Information files will be freely available online, and any third party is permitted to access, download, copy, distribute, and use these materials in any way, even commercially, with proper attribution. For more information, see our copyright guidelines: http://journals.plos.org/plosone/s/licenses-and-copyright.

We require you to either (1) present written permission from the copyright holder to publish these figures specifically under the CC BY 4.0 license, or (2) remove the figures from your submission:

1. You may seek permission from the original copyright holder of Figure(s) [#] to publish the content specifically under the CC BY 4.0 license.

We recommend that you contact the original copyright holder with the Content Permission Form (http://journals.plos.org/plosone/s/file?id=7c09/content-permission-form.pdf) and the following text:

“I request permission for the open-access journal PLOS ONE to publish XXX under the Creative Commons Attribution License (CCAL) CC BY 4.0 (http://creativecommons.org/licenses/by/4.0/). Please be aware that this license allows unrestricted use and distribution, even commercially, by third parties. Please reply and provide explicit written permission to publish XXX under a CC BY license and complete the attached form.”

Please upload the completed Content Permission Form or other proof of granted permissions as an "Other" file with your submission.

In the figure caption of the copyrighted figure, please include the following text: “Reprinted from [ref] under a CC BY license, with permission from [name of publisher], original copyright [original copyright year].”

2. If you are unable to obtain permission from the original copyright holder to publish these figures under the CC BY 4.0 license or if the copyright holder’s requirements are incompatible with the CC BY 4.0 license, please either i) remove the figure or ii) supply a replacement figure that complies with the CC BY 4.0 license. Please check copyright information on all replacement figures and update the figure caption with source information. If applicable, please specify in the figure caption text when a figure is similar but not identical to the original image and is therefore for illustrative purposes only.

5. If the reviewer comments include a recommendation to cite specific previously published works, please review and evaluate these publications to determine whether they are relevant and should be cited. There is no requirement to cite these works unless the editor has indicated otherwise.

[Note: HTML markup is below. Please do not edit.]

Reviewers' comments:

Reviewer's Responses to Questions

**Comments to the Author**

1. Is the manuscript technically sound, and do the data support the conclusions?

Reviewer #1: Yes

Reviewer #2: Yes

**********

2. Has the statistical analysis been performed appropriately and rigorously?

Reviewer #1: Yes

Reviewer #2: No

**********

3. Have the authors made all data underlying the findings in their manuscript fully available?

The PLOS Data policy

Reviewer #1: Yes

Reviewer #2: Yes

**********

4. Is the manuscript presented in an intelligible fashion and written in standard English?

Reviewer #1: Yes

Reviewer #2: No

**********

Reviewer #1: This manuscript investigates predictive factors for the development of sunset glow fundus (SGF) in patients with Vogt–Koyanagi–Harada (VKH) disease, focusing on parameters measured at 1 month after pulse steroid therapy. The study includes a relatively well-defined cohort of treatment-naïve patients with at least 12 months of follow-up, and the attempt to identify early predictive indicators is clinically relevant. However, several conceptual and methodological concerns limit the validity of the conclusions. In particular, the definition and timing of SGF assessment raise important issues that must be addressed before the manuscript can be considered for publication.

1) The most critical concern relates to the diagnostic definition and temporal interpretation of sunset glow fundus. In this study, patients were classified into SGF and non-SGF groups based on fundus findings at 12 months after treatment initiation. However, the manuscript implicitly assumes that SGF is a clearly definable endpoint within this timeframe and that parameters measured as early as 1 month can reliably predict its development. This assumption is problematic. The development of sunset glow fundus represents a chronic depigmentary process associated with sustained choroidal inflammation and melanocyte loss, and it typically evolves gradually over time. In clinical practice, the diagnosis of true sunset glow fundus often requires longer-term observation beyond 12 months, particularly in patients who receive aggressive early immunosuppressive therapy. Therefore, categorizing patients as having or not having SGF at 12 months may lead to misclassification, especially in cases with delayed or subclinical progression.

2) It is even more problematic to conceptually link parameters measured at 1 month to the definitive occurrence of sunset glow fundus. At 1 month after treatment initiation, patients are still in the early recovery phase of acute VKH, and inflammatory activity may fluctuate depending on treatment response, tapering schedule, and individual immune dynamics. It is therefore unrealistic to assume that a single measurement at this time point can serve as a reliable predictor of a chronic structural outcome such as sunset glow fundus. The temporal gap between early inflammatory changes and late depigmentary manifestations is biologically complex and cannot be reduced to a simple predictive relationship without careful longitudinal validation.

3) The manuscript does not provide a sufficiently rigorous definition of sunset glow fundus. It is unclear whether the diagnosis was based solely on color fundus photography, clinical judgment, or standardized criteria incorporating multimodal imaging such as fundus autofluorescence or indocyanine green angiography. Without explicit diagnostic criteria, the reliability and reproducibility of SGF classification remain uncertain. This is particularly important given that subtle depigmentary changes may be misinterpreted or overlooked, especially in the early stages.

4) Another issue relates to the statistical interpretation of the predictive model. The reported Youden index values for both BCVA and corneal thickness are below 0.3, indicating limited discriminatory ability. Indeed, the sensitivity values for both predictors are less than 0.5, as shown in Table 3 of the manuscript. These findings suggest that the proposed predictors have weak clinical utility, and the manuscript should explicitly acknowledge this limitation. Presenting these parameters as “key risk factors” may overstate their practical relevance.

5) The biological plausibility of corneal thickness as a predictor of sunset glow fundus also requires further discussion. While the authors suggest that corneal morphology may reflect inflammatory activity, the mechanistic link between corneal thickness and choroidal melanocyte loss is indirect at best. It is possible that corneal thickness represents a nonspecific marker of systemic or ocular inflammation rather than a disease-specific predictor. This distinction should be clarified.

6) Another important issue relates to the influence of treatment intensity on the long-term course of Vogt–Koyanagi–Harada (VKH) disease and the development of chronic depigmentary changes such as sunset glow fundus. The current manuscript focuses on predictive factors measured at 1 month after pulse steroid therapy; however, it does not sufficiently discuss how the initial treatment regimen itself may affect disease outcomes. It is now well recognized that the adequacy of early immunosuppression plays a critical role in preventing chronic evolution of VKH. In this context, the authors should cite and discuss the recent study by Tsubota et al., which directly compared the efficacy and safety of full-dose pulse versus half-pulse steroid therapy in VKH disease (Tsubota K, et al. Comparative study of efficacy and safety of pulse versus half-pulse steroid therapy for Vogt-Koyanagi-Harada Disease. Jpn J Ophthalmol. 2025;69:805–812). That study demonstrated that differences in initial steroid dosing strategies can significantly influence clinical outcomes, highlighting the importance of treatment-related variables in determining long-term disease progression.

Failure to account for variations in treatment intensity may confound the interpretation of predictive factors identified in the present study. Therefore, incorporation of this reference into the Introduction and Discussion sections would strengthen the scientific context and improve the clinical relevance of the manuscript.

7) From a clinical standpoint, the study would be strengthened by extending the follow-up period and evaluating SGF development at later time points, such as 18 or 24 months. Alternatively, the authors could redefine the outcome as “early depigmentary change” or “risk of chronic evolution” rather than definitive sunset glow fundus. Such terminology would better reflect the biological trajectory of VKH disease.

8) The manuscript would benefit from a clearer discussion of treatment-related variables. The tapering schedule of corticosteroids, the use of additional immunosuppressive agents, and the timing of therapy escalation are all factors known to influence chronic progression and SGF development. Without accounting for these variables, it is difficult to interpret the observed associations.

Reviewer #2: Despite the merit of this manuscript, the current form has multiple flaws should therefore be revised before it can be accepted.

Youden Index Discrepancies: The Abstract states that the Youden index for both BCVA and corneal thickness is 0.291. However, Table 3 reports a Youden index of 0.244 for corneal thickness. These values must be reconciled.

Contradictory Significance Labels: In the Results section, the authors report that RFT and CFT decreased significantly (p<0.001). Conversely, the legends for Figure 2 (k and l) state these changes occurred "without significant differences." This contradiction undermines the credibility of the findings.

Failure to Account for Inter-eye Correlation: The study analyzes 121 eyes from 61 patients. Because the two eyes of a single patient are highly correlated, treating them as independent samples in t-tests or ANOVA is statistically inappropriate. The authors must re-analyze the data using Generalized Estimating Equations (GEE) or Linear Mixed Models (LMM) to account for this clustering.

Logical Flaw in Exclusion Criteria: The manuscript lists "high myopia (greater than -8 diopters)" as an exclusion criterion. Clinically and mathematically, high myopia involves values less than -8D (e.g., -9D, -10D). Using "greater than" implies the exclusion of mild myopia, which appears to be a typographical or logical error.

Redundancy: In the multivariable analysis description, "ACV" (anterior chamber volume) is listed twice.

**********

what does this mean?). If published, this will include your full peer review and any attached files.

If you choose “no”, your identity will remain anonymous but your review may still be made public.

**Do you want your identity to be public for this peer review?**  For information about this choice, including consent withdrawal, please see our Privacy Policy

Reviewer #1: No

Reviewer #2: No

**********

[NOTE: If reviewer comments were submitted as an attachment file, they will be attached to this email and accessible via the submission site. Please log into your account, locate the manuscript record, and check for the action link "View Attachments". If this link does not appear, there are no attachment files.]

To ensure your figures meet our technical requirements, please review our figure guidelines: https://journals.plos.org/plosone/s/figures

You may also use PLOS’s free figure tool, NAAS, to help you prepare publication quality figures: https://journals.plos.org/plosone/s/figures#loc-tools-for-figure-preparation.

NAAS will assess whether your figures meet our technical requirements by comparing each figure against our figure specifications.

# Author comment: Author response to Decision Letter 1

**DOI:** 10.1371/journal.pone.0362558.r002

17 Jun 2026

Journal Requirements:

When submitting your revision, we need you to address these additional requirements.

1. Please ensure that your manuscript meets PLOS ONE's style requirements, including those for file naming. The PLOS ONE style templates can be found at

https://journals.plos.org/plosone/s/file?id=wjVg/PLOSOne_formatting_sample_main_body.pdf and

https://journals.plos.org/plosone/s/file?id=ba62/PLOSOne_formatting_sample_title_authors_affiliations.pdf

Yes, we revised headings in Bold type, 18pt font.

2. Thank you for stating the following in the Acknowledgments Section of your manuscript:

“The authors received no external funding or financial support.”

We note that you have provided funding information that is not currently declared in your Funding Statement. However, funding information should not appear in the Acknowledgments section or other areas of your manuscript. We will only publish funding information present in the Funding Statement section of the online submission form.

Please remove any funding-related text from the manuscript and let us know how you would like to update your Funding Statement. Currently, your Funding Statement reads as follows:

“The author(s) received no specific funding for this work.”

Please include your amended statements within your cover letter; we will change the online submission form on your behalf.

Yes, we removed funding-related text from the manuscript as below.

Funding declaration

The authors received no external funding or financial support.

3. Please include a separate caption for each figure in your manuscript.

Yes, we included a separate caption for each figure in our manuscript.

4. We note that Figure 1 in your submission contain copyrighted images. All PLOS content is published under the Creative Commons Attribution License (CC BY 4.0), which means that the manuscript, images, and Supporting Information files will be freely available online, and any third party is permitted to access, download, copy, distribute, and use these materials in any way, even commercially, with proper attribution. For more information, see our copyright guidelines: http://journals.plos.org/plosone/s/licenses-and-copyright.

We require you to either (1) present written permission from the copyright holder to publish these figures specifically under the CC BY 4.0 license, or (2) remove the figures from your submission:

Yes, we removed the figure 1 from our submission. According to this revision, we deleted a below paragraph from results.

Fig. 1 illustrates PentacamⓇ measurements at 1 month and color fundus images at 12 months post-treatment for the SGF and non-SGF groups. Increased corneal thickness, as indicated by “Pachy” in the figures, is observed in association with SGF development.

1. You may seek permission from the original copyright holder of Figure(s) [#] to publish the content specifically under the CC BY 4.0 license.

We recommend that you contact the original copyright holder with the Content Permission Form (http://journals.plos.org/plosone/s/file?id=7c09/content-permission-form.pdf) and the following text:

“I request permission for the open-access journal PLOS ONE to publish XXX under the Creative Commons Attribution License (CCAL) CC BY 4.0 (http://creativecommons.org/licenses/by/4.0/). Please be aware that this license allows unrestricted use and distribution, even commercially, by third parties. Please reply and provide explicit written permission to publish XXX under a CC BY license and complete the attached form.”

Please upload the completed Content Permission Form or other proof of granted permissions as an "Other" file with your submission.

In the figure caption of the copyrighted figure, please include the following text: “Reprinted from [ref] under a CC BY license, with permission from [name of publisher], original copyright [original copyright year].”

2. If you are unable to obtain permission from the original copyright holder to publish these figures under the CC BY 4.0 license or if the copyright holder’s requirements are incompatible with the CC BY 4.0 license, please either i) remove the figure or ii) supply a replacement figure that complies with the CC BY 4.0 license. Please check copyright information on all replacement figures and update the figure caption with source information. If applicable, please specify in the figure caption text when a figure is similar but not identical to the original image and is therefore for illustrative purposes only.

5. If the reviewer comments include a recommendation to cite specific previously published works, please review and evaluate these publications to determine whether they are relevant and should be cited. There is no requirement to cite these works unless the editor has indicated otherwise.

[Note: HTML markup is below. Please do not edit.]

Reviewers' comments:

Reviewer's Responses to Questions

Comments to the Author

1. Is the manuscript technically sound, and do the data support the conclusions?

The manuscript must describe a technically sound piece of scientific research with data that supports the conclusions. Experiments must have been conducted rigorously, with appropriate controls, replication, and sample sizes. The conclusions must be drawn appropriately based on the data presented.

Reviewer #1: Yes

Reviewer #2: Yes

________________________________________

2. Has the statistical analysis been performed appropriately and rigorously?

Reviewer #1: Yes

Reviewer #2: No

________________________________________

3. Have the authors made all data underlying the findings in their manuscript fully available?

The PLOS Data policy requires authors to make all data underlying the findings described in their manuscript fully available without restriction, with rare exception (please refer to the Data Availability Statement in the manuscript PDF file). The data should be provided as part of the manuscript or its supporting information, or deposited to a public repository. For example, in addition to summary statistics, the data points behind means, medians and variance measures should be available. If there are restrictions on publicly sharing data—e.g. participant privacy or use of data from a third party—those must be specified.

Reviewer #1: Yes

Reviewer #2: Yes

________________________________________

4. Is the manuscript presented in an intelligible fashion and written in standard English?

PLOS ONE does not copyedit accepted manuscripts, so the language in submitted articles must be clear, correct, and unambiguous. Any typographical or grammatical errors should be corrected at revision, so please note any specific errors here.

Reviewer #1: Yes

Reviewer #2: No

________________________________________

5. Review Comments to the Author

Please use the space provided to explain your answers to the questions above. You may also include additional comments for the author, including concerns about dual publication, research ethics, or publication ethics. (Please upload your review as an attachment if it exceeds 20,000 characters)

Reviewer #1: This manuscript investigates predictive factors for the development of sunset glow fundus (SGF) in patients with Vogt–Koyanagi–Harada (VKH) disease, focusing on parameters measured at 1 month after pulse steroid therapy. The study includes a relatively well-defined cohort of treatment-naïve patients with at least 12 months of follow-up, and the attempt to identify early predictive indicators is clinically relevant. However, several conceptual and methodological concerns limit the validity of the conclusions. In particular, the definition and timing of SGF assessment raise important issues that must be addressed before the manuscript can be considered for publication.

1) The most critical concern relates to the diagnostic definition and temporal interpretation of sunset glow fundus. In this study, patients were classified into SGF and non-SGF groups based on fundus findings at 12 months after treatment initiation. However, the manuscript implicitly assumes that SGF is a clearly definable endpoint within this timeframe and that parameters measured as early as 1 month can reliably predict its development. This assumption is problematic. The development of sunset glow fundus represents a chronic depigmentary process associated with sustained choroidal inflammation and melanocyte loss, and it typically evolves gradually over time. In clinical practice, the diagnosis of true sunset glow fundus often requires longer-term observation beyond 12 months, particularly in patients who receive aggressive early immunosuppressive therapy. Therefore, categorizing patients as having or not having SGF at 12 months may lead to misclassification, especially in cases with delayed or subclinical progression.

Yes, we made a below paragraph in discussion.

The present study has several limitations. First, patients were classified into the SGF and non-SGF groups on the basis of fundus findings 12 months after treatment initiation. In clinical practice, a definitive diagnosis of SGF often requires observation beyond 12 months, particularly in patients who receive aggressive early immunosuppressive therapy. Therefore, classification of the SGF and non-SGF groups based on fundus findings obtained at a later time point may be more appropriate. Second, we assessed predictive factors at 1 month after pulse steroid therapy. At this stage, patients are still in the early recovery phase of acute VKH disease, and inflammatory activity may fluctuate depending on treatment response, the tapering schedule, and individual immune dynamics. Therefore, it may be unrealistic to assume that a single measurement obtained at this time point can serve as a reliable predictor of a chronic structural outcome such as SGF. Third, the Youden index values for both BCVA and corneal thickness were < 0.3, and the sensitivity values for both predictors were < 0.5, as shown in Table 3. These findings suggest that the proposed predictors have limited clinical utility. Fourth, corneal thickness may represent a nonspecific marker of systemic or ocular inflammation rather than a disease-specific predictor. It was not possible to distinguish between these possibilities in the present study.

Furthermore, we revised title and short title as below.

Predictive factors for risk of chronic evolution in Vogt–Koyanagi–Harada disease at 1 month after pulse steroid therapy

Short title: Predictive factors for risk of chronic evolution

2) It is even more problematic to conceptually link parameters measured at 1 month to the definitive occurrence of sunset glow fundus. At 1 month after treatment initiation, patients are still in the early recovery phase of acute VKH, and inflammatory activity may fluctuate depending on treatment response, tapering schedule, and individual immune dynamics. It is therefore unrealistic to assume that a single measurement at this time point can serve as a reliable predictor of a chronic structural outcome such as sunset glow fundus. The temporal gap between early inflammatory changes and late depigmentary manifestations is biologically complex and cannot be reduced to a simple predictive relationship without careful longitudinal validation.

Yes, we made a below paragraph in discussion.

The present study has several limitations. First, patients were classified into the SGF and non-SGF groups on the basis of fundus findings 12 months after treatment initiation. In clinical practice, a definitive diagnosis of SGF often requires observation beyond 12 months, particularly in patients who receive aggressive early immunosuppressive therapy. Therefore, classification of the SGF and non-SGF groups based on fundus findings obtained at a later time point may be more appropriate. Second, we assessed predictive factors at 1 month after pulse steroid therapy. At this stage, patients are still in the early recovery phase of acute VKH disease, and inflammatory activity may fluctuate depending on treatment response, the tapering schedule, and individual immune dynamics. Therefore, it may be unrealistic to assume that a single measurement obtained at this time point can serve as a reliable predictor of a chronic structural outcome such as SGF. Third, the Youden index values for both BCVA and corneal thickness were < 0.3, and the sensitivity values for both predictors were < 0.5, as shown in Table 3. These findings suggest that the proposed predictors have limited clinical utility. Fourth, corneal thickness may represent a nonspecific marker of systemic or ocular inflammation rather than a disease-specific predictor. It was not possible to distinguish between these possibilities in the present study.

3) The manuscript does not provide a sufficiently rigorous definition of sunset glow fundus. It is unclear whether the diagnosis was based solely on color fundus photography, clinical judgment, or standardized criteria incorporating multimodal imaging such as fundus autofluorescence or indocyanine green angiography. Without explicit diagnostic criteria, the reliability and reproducibility of SGF classification remain uncertain. This is particularly important given that subtle depigmentary changes may be misinterpreted or overlooked, especially in the early stages.

Yes, we added below sentences in materials and methods.

A single investigator (TM) assessed the ocular findings at approximately monthly intervals, and routinely determined the onset of SGF based on the appearance of the orange-red fundus discoloration that had not been present at the visit 1 month earlier. The presence of SGF in each eye was subsequently reconfirmed by reviewing the color fundus photographs.

4) Another issue relates to the statistical interpretation of the predictive model. The reported Youden index values for both BCVA and corneal thickness are below 0.3, indicating limited discriminatory ability. Indeed, the sensitivity values for both predictors are less than 0.5, as shown in Table 3 of the manuscript. These findings suggest that the proposed predictors have weak clinical utility, and the manuscript should explicitly acknowledge this limitation. Presenting these parameters as “key risk factors” may overstate their practical relevance.

Yes, we revised as below in abstract.

Elevated logarithmic BCVA values and increased corneal thickness at 1 month after treatment were identified as key risk factors for the subsequent SGF development.

Furthermore, we made a below paragraph in discussion.

The present study has several limitations. First, patients were classified into the SGF and non-SGF groups on the basis of fundus findings 12 months after treatment initiation. In clinical practice, a definitive diagnosis of SGF often requires observation beyond 12 months, particularly in patients who receive aggressive early immunosuppressive therapy. Therefore, classification of the SGF and non-SGF groups based on fundus findings obtained at a later time point may be more appropriate. Second, we assessed predictive factors at 1 month after pulse steroid therapy. At this stage, patients are still in the early recovery phase of acute VKH disease, and inflammatory activity may fluctuate depending on treatment response, the tapering schedule, and individual immune dynamics. Therefore, it may be unrealistic to assume that a single measurement obtained at this time point can serve as a reliable predictor of a chronic structural outcome such as SGF. Third, the Youden index values for both BCVA and corneal thickness were < 0.3, and the sensitivity values for both predictors were < 0.5, as shown in Table 3. These findings suggest that the proposed predictors have limited clinical utility. Fourth, corneal thickness may represent a nonspecific marker of systemic or ocular inflammation rather than a disease-specific predictor. It was not possible to distinguish between these possibilities in the present study.

5) The biological plausibility of corneal thickness as a predictor of sunset glow fundus also requires further discussion. While the authors suggest that corneal morphology may reflect inflammatory activity, the mechanistic link between corneal thickness and choroidal melanocyte loss is indirect at best. It is possible that corneal thickness represents a nonspecific marker of systemic or ocular inflammation rather than a disease-specific predictor. This distinction should be clarified.

Yes, we made a below paragraph in discussion.

The present study has several limitations. First, patients were classified into the SGF and non-SGF groups on the basis of fundus findings 12 months after treatment initiation. In clinical practice, a definitive diagnosis of SGF often requires observation beyond 12 months, particularly in patients who receive aggressive early immunosuppressive therapy. Therefore, classification of the SGF and non-SGF groups based on fundus findings obtained at a later time point may be more appropriate. Second, we assessed predictive factors at 1 month after pulse steroid therapy. At this stage, patients are still in the early recovery phase of acute VKH disease, and inflammatory activity may fluctuate depending on treatment response, the tapering schedule, and individual immune dynamics. Therefore, it may be unrealistic to assume that a single measurement obtained at this time point can serve as a reliable predictor of a chronic structural outcome such as SGF. Third, the Youden index values for both BCVA and corneal thickness were < 0.3, and the sensitivity values for both predictors were < 0.5, as shown in Table 3. These findings suggest that the proposed predictors have limited clinical utility. Fourth, corneal thickness may represent a nonspecific marker of systemic or ocular inflammation rather than a disease-specific predictor. It was not possible to distinguish between these possibilities in the present study.

6) Another important issue relates to the influence of treatment intensity on the long-term course of Vogt–Koyanagi–Harada (VKH) disease and the development of chronic depigmentary changes such as sunset glow fundus. The current manuscript focuses on predictive factors measured at 1 month after pulse steroid therapy; however, it does not sufficiently discuss how the initial treatment regimen itself may affect disease outcomes. It is now well recognized that the adequacy of early immunosuppression plays a critical role in preventing chronic evolution of VKH. In this context, the authors should cite and discuss the recent study by Tsubota et al., which directly compared the efficacy and safety of full-dose pulse versus half-pulse steroid therapy in VKH disease (Tsubota K, et al. Comparative study of efficacy and safety of pulse versus half-pulse steroid therapy for Vogt-Koyanagi-Harada Disease. Jpn J Ophthalmol. 2025;69:805–812). That study demonstrated that differences in initial steroid dosing strategies can significantly influence clinical outcomes, highlighting the importance of treatment-related variables in determining long-term disease progression.

Failure to account for variations in treatment intensity may confound the interpretation of predictive factors identified in the present study. Therefore, incorporation of this reference into the Introduction and Discussion sections would strengthen the scientific context and improve the clinical relevance of the manuscript.

Yes, we added below paper as reference no.8. According to this revision, Introduction was revised as below.

8. Tsubota K, Goto H, Asakage M, Kinoshita Y, Nonaka R, Wakatsuki K, et al. Comparative study of efficacy and safety of pulse versus half pulse steroid therapy for Vogt–Koyanagi–Harada disease. Jpn J Ophthalmol. 2025;69:805–812. doi: 10.1007/s10384-025-01213-3.

Vogt–Koyanagi–Harada (VKH) disease is a bilateral granulomatous panuveitis with potential multisystem involvement. It is a T-cell-mediated autoimmune disorder that occurs in genetically susceptible individuals, in which cytotoxic T-cells target melanocytes [1]. As melanocytes are widely distributed throughout the body, systemic manifestations such as fever meningitis-like symptoms, headache, paresthesia of hair, and general malaise often precede the onset of ocular symptoms. The disease can develop across a wide age range, from infancy to older adulthood, but is reported more frequently in females [2-8]. Adequate inhibition of inflammation during the early stage of the disease is important for preventing recurrence and the persistence of inflammation during later progressive stages.

In Japan, corticosteroid pulse therapy consisting of 500–1000 mg of intravenous methylprednisolone administered over 3 days, followed by several months of oral corticosteroids, has been widely implemented for many years [2-8].

Furthermore, we added a below paragraph in discussion.

It is now well recognized that adequate of early immunosuppression plays a critical role in preventing the chronic evolution of VKH disease. Tsubota et al. reported no significant difference in clinical efficacy between conventional pulse steroid therapy (1,000 mg/day of intravenous methylprednisolone for 3 days) and half-pulse steroid therapy (500 mg/day of intravenous methylprednisolone for 3 days) for VKH disease [8], concluding that half-pulse steroid therapy may reduce the risk of corticosteroid-related adverse effects [8]. They demonstrated that differences in initial steroid dosing strategies can significantly influence clinical outcomes, highlighting the importance of treatment-related variables in determining long-term disease progression [8]. Because pulse steroid therapy is associated with several adverse effects [21], their study is noteworthy in suggesting a strategy to reduce this risk [8].

7) From a clinical standpoint, the study would be strengthened by extending the follow-up period and evaluating SGF development at later time points, such as 18 or 24 months. Alternatively, the authors could redefine the outcome as “early depigmentary change” or “risk of chronic evolution” rather than definitive sunset glow fundus. Such terminology would better reflect the biological trajectory of VKH disease.

Yes, we revised title and short title as below.

Predictive factors for risk of chronic evolution in Vogt–Koyanagi–Harada disease at 1 month after pulse steroid therapy

Short title: Predictive factors for risk of chronic evolution

We revised last paragraph in introduction as below.

The aim of this study was to identify factors quantifiable at 1 month after pulse steroid therapy, as well as at the initial patient visit, that could predict the risk of chronic evolution 12 months post-treatment in patients with acute VKH disease.

Furthermore, we revised last paragraph in discussion as below.

In conclusion, this study identified two risk factors at 1 month post-treatment, along with their corresponding cut-off values, that are associated with the risk of chronic evolution. These findings may support more informed clinical decision-making in patients with VKH disease, particularly those presenting with BCVA and corneal thickness values exceeding the identified thresholds. Our future objectives include identifying more reliable predictive factors and establishing management strategies for cases exceeding the identified cut-off values.

8) The manuscript would benefit from a clearer discussion of treatment-related variables. The tapering schedule of corticosteroids, the use of additional immunosuppressive agents, and the timing of therapy escalation are all factors known to influence chronic progression and SGF development. Without accounting for these variables, it is difficult to interpret the observed associations.

Yes, we revised materials and methods as below.

Pulse steroid therapy was initiated with intravenous methylprednisolone (Solu-Medrol; Pfizer, NY, USA) at a dose of 1,000 mg/day for 3 days, followed by oral prednisolone (Predonine; Shionogi, Osaka, Japan). Oral prednisolone was started at 40 mg/day and tapered by 5 mg/day every 3 − 4 weeks, as previously described [2, 3]. Although one patient was 13 years old, her body weight (48 kg) allowed for the administration of the standard steroid regimen. Patients with recurrence limited to the anterior segment were treated with 0.1% fluorometholone ophthalmic suspension (Santen, Osaka, Japan) or Sanbetason ophthalmic and otorhinologic solution (Santen, Osaka, Japan). Patients with recurrence in the posterior segment were treated with oral corticosteroids or sub-Tenon triamcinolone acetonide injections. If anterior inflammation progressed to the chronic type and became resistant to steroid therapy, adalimumab was administered.

Reviewer #2: Despite the merit of this manuscript, the current form has multiple flaws should therefore be revised before it can be accepted.

Youden Index Discrepancies: The Abstract states that the Youden index for both BCVA and corneal thickness is 0.291. However, Table 3 reports a Youden index of 0.244 for corneal thickness. These values must be reconciled.

Yes, we revised a numerical value in abstract as below.

The corresponding Youden index values were 0.291 for BCVA and 0.244 for corneal thickness.

Contradictory Significance Labels: In the Results section, the authors report that RFT and CFT decreased significantly (p<0.001). Conversely, the legends for Figure 2 (k and l) state these changes occurred "without significant differences." This contradiction undermines the credibility of the findings.

Yes, we revised in the results section as below.

Both RFT and CFT decreased significantly over the observation period in both groups according to one-way repeated-measures ANOVA (p < 0.001) (Fig. 1 k, l).

We revised the legends for Figure 1 (k and l) as below.

(k) RFT at pretreatment and at 1, 6, and 12 months post-treatment, without significant differences according to the unpaired t-test. (l) CFT at pretreatment and at 1, 6, and 12 months post-treatment, with significant differences at pretreatment (p < 0.01), and 1 month (p < 0.05) according to the unpaired t-test.

Failure to Account for Inter-eye Correlation: The study analyzes 121 eyes from 61 patients. Because the two eyes of a single patient are highly correlated, treating them as independent samples in t-tests or ANOVA is statistically inappropriate. The authors must re-analyze the data using Generalized Estimating Equations (GEE) or Linear Mixed Models (LMM) to account for this clustering.

Yes, we re-analyzed the data using Generalized Estimating Equations (GEE) and made new Figure 2 (a~l).

Fig. 2 illustrates the chronological changes in mean biomarker values in both groups, as analyzed using GEEs. BCVA improved significantly over time in both groups (Fig. 2a). ACV (Fig. 2b) and central ACD values (Fig. 2c) also increased significantly over time in both groups. Peripheral ACD showed a similar pattern (Fig. 2d). ACA increased significantly in the non-SGF group remained largely unchanged in the SGF group (Fig. 2e). Pupil diameter (Fig. 2f) and corneal thickness (Fig. 2g) remained unchanged over time in both groups. SE decreased significantly in the SGF group, but showed minimal change in the non-SGF group (Fig. 2h). AL increased significantly in both groups (Fig. 2i). Lens thickness decreased significantly over time in the non-SGF group, but remained stable in the SGF group (Fig. 2j). Both RFT and CFT decreased significantly over the observation period in both groups according to the GEE analysis (p < 0.001) (Fig. 2k, l).

Fig. 2.

(a) Longitudinal changes in BCVA, as the logarithm of the minimum angle of resolution

for the SGF and non-SGF groups at pretreatment and at 1, 6, and 12 months post-treatment. A significant difference was observed at 1 month (p < 0.01). (b) ACV at pretreatment and at 1, 6, and 12 months post-treatment. without significant differences. (c) Central ACD at pretreatment and at 1, 6, and 12 months post-treatment. No significant differences were observed. (d) Peripheral ACD at pretreatment and at 1, 6, and 12 months post-treatment. No significant differences were observed. (e) ACA at pretreatment and at 1, 6, and 12 months post-treatment. No significant differences were observed. (f) Pupil diameter at pretreatment and at 1, 6, and 12 months post-treatment. No significant differences were observed. (g) Corneal thickness at pretreatment and at 1, 6, and 12 months post-treatment. Significant differences were observed at pretreatment (p < 0.001). (h) SE at pretreatment and at 1, 6, and 12 months post-treatment. No significant differences were observed. (i) AL at pretreatment and at 1, 6, and 12 months post-treatment. No significant differences were observed. (j) Lens thickness at pretreatment and at 1, 6, and 12 months post-treatment. No significant differences were observed. (k) RFT at pretreatment and at 1, 6, and 12 months post-treatment. No significant differences were observed. (l) CFT at pretreatment and at 1, 6, and 12 months post-treatment. No significant differences were observed.

Abbreviations: ACA, anterior chamber angle; ACD, anterior chamber depth; ACV, anterior chamber volume; AL, axial length; BCVA, best-corrected visual acuity; CFT, choroidal foveal thickness; RFT, retinal foveal thickness; SE, spherical equivalent.

Logical Flaw in Exclusion Criteria: The manuscript lists "high myopia (greater than -8 diopters)" as an exclusion criterion. Clinically and mathematically, high myopia involves values less than -8D (e.g., -9D, -10D). Using "greater than" implies the exclusion of mild myopia, which appears to be a typographical or logical error.

Yes, we revised as below.

high myopia (less than -8 diopters)

Redundancy: In the multivariable analysis description, "ACV" (anterior chamber volume) is listed twice.

Yes, we revised as below.

These outcomes included BCVA, ACV, ACD (also peripheral), corneal thickness, SE and changes in these parameters from the initial visit to 1 month, ACV, difference of ACV from initial visit to 1 month post-treatment, as well as ACA, pupil diameter, AL, RFT, and CFT.

________________________________________

6. PLOS authors have the option to publish the peer review history of their article (what does this mean?). If published, this will include your full peer review and any attached files.

If you choose “no”, your identity will remain anonymous but your review may still be made public.

Do you want your identity to be public for this peer review? For information about this choice, including consent withdrawal, please see our Privacy Policy.

Reviewer #1: No

Reviewer #2: No

________________________________________

Attachment Submitted filename: Response to Reviewers.docx

# Peer review: Decision Letter 1

**DOI:** 10.1371/journal.pone.0362558.r003

**Authors:** Robin Haunschild

8 Sep 2026

Dear Dr. Muto,

Thank you for submitting your manuscript to PLOS One. After careful consideration, we feel that it has merit but does not fully meet PLOS One’s publication criteria as it currently stands. Therefore, we invite you to submit a revised version of the manuscript that addresses the points raised during the review process.

Please submit your revised manuscript by Oct 23 2026 11:59PM. If you will need more time than this to complete your revisions, please reply to this message or contact the journal office at plosone@plos.org. When you're ready to submit your revision, log on to https://www.editorialmanager.com/pone/ and select the 'Submissions Needing Revision' folder to locate your manuscript file.

- A letter that responds to each point raised by the academic editor and reviewer(s). You should upload this letter as a separate file labeled 'Response to Reviewers'.
- A marked-up copy of your manuscript that highlights changes made to the original version. You should upload this as a separate file labeled 'Revised Manuscript with Track Changes'.
- An unmarked version of your revised paper without tracked changes. You should upload this as a separate file labeled 'Manuscript'.

If you would like to make changes to your financial disclosure, please include your updated statement in your cover letter. Guidelines for resubmitting your figure files are available below the reviewer comments at the end of this letter.

If applicable, we recommend that you deposit your laboratory protocols in protocols.io to enhance the reproducibility of your results. Protocols.io assigns your protocol its own identifier (DOI) so that it can be cited independently in the future. For instructions see: https://journals.plos.org/plosone/s/submission-guidelines#loc-laboratory-protocols. Additionally, PLOS One offers an option for publishing peer-reviewed Lab Protocol articles, which describe protocols hosted on protocols.io. Read more information on sharing protocols at https://plos.org/protocols?utm_medium=editorial-email&utm_source=authorletters&utm_campaign=protocols.

As the corresponding author, your ORCID iD is verified in the submission system and will appear in the published article. PLOS supports the use of ORCID, and we encourage all coauthors to register for an ORCID iD and use it as well. Please encourage your coauthors to verify their ORCID iD within the submission system before final acceptance, as unverified ORCID iDs will not appear in the published article. *Only*  the individual author can complete the verification step; PLOS staff *cannot*  verify ORCID iDs on behalf of authors.

We look forward to receiving your revised manuscript.

Kind regards,

Robin Haunschild

Academic Editor

PLOS One

Journal Requirements:

If the reviewer comments include a recommendation to cite specific previously published works, please review and evaluate these publications to determine whether they are relevant and should be cited. There is no requirement to cite these works unless the editor has indicated otherwise.

[Note: HTML markup is below. Please do not edit.]

Reviewers' comments:

Reviewer's Responses to Questions

**Comments to the Author**

Reviewer #2: All comments have been addressed

Reviewer #3: (No Response)

**********

2. Is the manuscript technically sound, and do the data support the conclusions?

Reviewer #2: Yes

Reviewer #3: Partly

**********

3. Has the statistical analysis been performed appropriately and rigorously?

Reviewer #2: Yes

Reviewer #3: Yes

**********

4. Have the authors made all data underlying the findings in their manuscript fully available?

The PLOS Data policy

Reviewer #2: Yes

Reviewer #3: Yes

**********

5. Is the manuscript presented in an intelligible fashion and written in standard English?

Reviewer #2: Yes

Reviewer #3: Yes

**********

Reviewer #2: The authors have addressed all the major issues of the original submission, and the current version is acceptable to be published.

Reviewer #3: Comments to the Authors:

The manuscript investigates early predictive factors for sunset glow fundus (SGF) development in patients with acute VKH disease. While the study addresses a clinically relevant question, several methodological and conceptual concerns must be addressed before the manuscript can be considered for publication.

Major Concerns:

1. Role of Extraocular Manifestations: The current study focuses exclusively on ocular parameters (anterior chamber morphology, BCVA, etc.). However, systemic manifestations, such as meningismus, tinnitus, dysacusis, and high sensitivity to touch of the hair, are frequently observed in VKH disease and have been strongly associated with disease progression. The authors should analyze the role of extraocular manifestations in their cohort as potential predictors for SGF. (PMID: 37058172; 17123618).

2. The manuscript does not clearly state which diagnostic criteria were used to classify the patients at the initial visit. While older criteria are traditionally common, recent literature has introduced updated diagnostic criteria for VKH disease that demonstrate significant advantages, notably a higher diagnostic sensitivity (PMID: 29978190). Please clarify the exact diagnostic criteria applied to this cohort in the Methods section.

3. The study cohort consists of 61 patients (121 eyes). It is unclear if this sample size provides sufficient statistical power to support the conclusions drawn from the multivariable analysis, particularly given the low sensitivity (<0.5) and Youden index (<0.3) values reported. Has a formal a priori sample size calculation or post hoc power analysis been performed? Please clarify this in the methodology.

**********

what does this mean?). If published, this will include your full peer review and any attached files.

If you choose “no”, your identity will remain anonymous but your review may still be made public.

**Do you want your identity to be public for this peer review?**  For information about this choice, including consent withdrawal, please see our Privacy Policy

Reviewer #2: No

Reviewer #3: No

**********

[NOTE: If reviewer comments were submitted as an attachment file, they will be attached to this email and accessible via the submission site. Please log into your account, locate the manuscript record, and check for the action link "View Attachments". If this link does not appear, there are no attachment files.]

To ensure your figures meet our technical requirements, please review our figure guidelines: https://journals.plos.org/plosone/s/figures

You may also use PLOS’s free figure tool, NAAS, to help you prepare publication quality figures: https://journals.plos.org/plosone/s/figures#loc-tools-for-figure-preparation.

NAAS will assess whether your figures meet our technical requirements by comparing each figure against our figure specifications.

# Author comment: Author response to Decision Letter 2

**DOI:** 10.1371/journal.pone.0362558.r004

15 Sep 2026

None.

Attachment Submitted filename: Response to reviewer.docx

# Peer review: Decision Letter 2

**DOI:** 10.1371/journal.pone.0362558.r005

**Authors:** Robin Haunschild

27 Oct 2026

Predictive factors for risk of chronic evolution in Vogt–Koyanagi–Harada disease at 1 month after pulse steroid therapy

PONE-D-26-10226R2

Dear Dr. Muto,

We’re pleased to inform you that your manuscript has been judged scientifically suitable for publication and will be formally accepted for publication once it meets all outstanding technical requirements.

Within one week, you’ll receive an e-mail detailing the required amendments. When these have been addressed, you’ll receive a formal acceptance letter and your manuscript will be scheduled for publication.

An invoice will be generated when your article is formally accepted. Please note, if your institution has a publishing partnership with PLOS and your article meets the relevant criteria, all or part of your publication costs will be covered. Please make sure your user information is up-to-date by logging into Editorial Manager at Editorial Manager® and clicking the ‘Update My Information' link at the top of the page. For questions related to billing, please contact billing support.

If your institution or institutions have a press office, please notify them about your upcoming paper to help maximize its impact. If they’ll be preparing press materials, please inform our press team as soon as possible -- no later than 48 hours after receiving the formal acceptance. Your manuscript will remain under strict press embargo until 2 pm Eastern Time on the date of publication. For more information, please contact onepress@plos.org.

Kind regards,

Robin Haunschild

Academic Editor

PLOS One

Additional Editor Comments (optional):

Reviewers' comments:

Reviewer's Responses to Questions

**Comments to the Author**

Reviewer #3: All comments have been addressed

**********

2. Is the manuscript technically sound, and do the data support the conclusions?

Reviewer #3: Yes

**********

3. Has the statistical analysis been performed appropriately and rigorously?

Reviewer #3: Yes

**********

4. Have the authors made all data underlying the findings in their manuscript fully available?

The PLOS Data policy

Reviewer #3: Yes

**********

5. Is the manuscript presented in an intelligible fashion and written in standard English?

Reviewer #3: Yes

**********

Reviewer #3: I have carefully reviewed the manuscript and provided all my detailed comments and suggestions in the previous sections. I do not have any additional comments to add at this time. I believe the authors will carefully consider and address the points raised above.

**********

what does this mean?). If published, this will include your full peer review and any attached files.

If you choose “no”, your identity will remain anonymous but your review may still be made public.

**Do you want your identity to be public for this peer review?**  For information about this choice, including consent withdrawal, please see our Privacy Policy

Reviewer #3: No

**********

# Editor report: Acceptance letter

**DOI:** 10.1371/journal.pone.0362558.r006

**Authors:** Robin Haunschild

PONE-D-26-10226R2

PLOS One

Dear Dr. Muto,

I'm pleased to inform you that your manuscript has been deemed suitable for publication in PLOS One. Congratulations! Your manuscript is now being handed over to our production team.

At this stage, our production department will prepare your paper for publication. This includes ensuring the following:

* All references, tables, and figures are properly cited

* All relevant supporting information is included in the manuscript submission,

* There are no issues that prevent the paper from being properly typeset

You will receive further instructions from the production team, including instructions on how to review your proof when it is ready. Please keep in mind that we are working through a large volume of accepted articles, so please give us a few days to review your paper and let you know the next and final steps.

Lastly, if your institution or institutions have a press office, please let them know about your upcoming paper now to help maximize its impact. If they'll be preparing press materials, please inform our press team within the next 48 hours. Your manuscript will remain under strict press embargo until 2 pm Eastern Time on the date of publication. For more information, please contact onepress@plos.org.

You will receive an invoice from PLOS for your publication fee after your manuscript has reached the completed accept phase. If you receive an email requesting payment before acceptance or for any other service, this may be a phishing scheme. Learn how to identify phishing emails and protect your accounts at https://explore.plos.org/phishing.

If we can help with anything else, please email us at customercare@plos.org.

Thank you for submitting your work to PLOS One and supporting open access.

Kind regards,

PLOS One Editorial Office Staff

on behalf of

Dr. Robin Haunschild

Academic Editor

PLOS One
